# How Do Autonomous Vehicles Decide?

**DOI:** 10.3390/s23010317

**Published:** 2022-12-28

**Authors:** Sumbal Malik, Manzoor Ahmed Khan, Hesham El-Sayed, Jalal Khan, Obaid Ullah

**Affiliations:** 1 College of Information Technology, United Arab Emirates University, Abu Dhabi 15551, United Arab Emirates; 2 Emirates Center for Mobility Research (ECMR), United Arab Emirates University, Abu Dhabi 15551, United Arab Emirates

**Keywords:** autonomous driving, decision-making, behavioural planning

## Abstract

The advancement in sensor technologies, mobile network technologies, and artificial intelligence has pushed the boundaries of different verticals, e.g., eHealth and autonomous driving. Statistics show that more than one million people are killed in traffic accidents yearly, where the vast majority of the accidents are caused by human negligence. Higher-level autonomous driving has great potential to enhance road safety and traffic efficiency. One of the most crucial links to building an autonomous system is the task of decision-making. The ability of a vehicle to make robust decisions on its own by anticipating and evaluating future outcomes is what makes it intelligent. Planning and decision-making technology in autonomous driving becomes even more challenging, due to the diversity of the dynamic environments the vehicle operates in, the uncertainty in the sensor information, and the complex interaction with other road participants. A significant amount of research has been carried out toward deploying autonomous vehicles to solve plenty of issues, however, how to deal with the high-level decision-making in a complex, uncertain, and urban environment is a comparatively less explored area. This paper provides an analysis of decision-making solutions approaches for autonomous driving. Various categories of approaches are analyzed with a comparison to classical decision-making approaches. Following, a crucial range of research gaps and open challenges have been highlighted that need to be addressed before higher-level autonomous vehicles hit the roads. We believe this survey will contribute to the research of decision-making methods for autonomous vehicles in the future by equipping the researchers with an overview of decision-making technology, its potential solution approaches, and challenges.

## 1. Introduction

**Figure 1:**
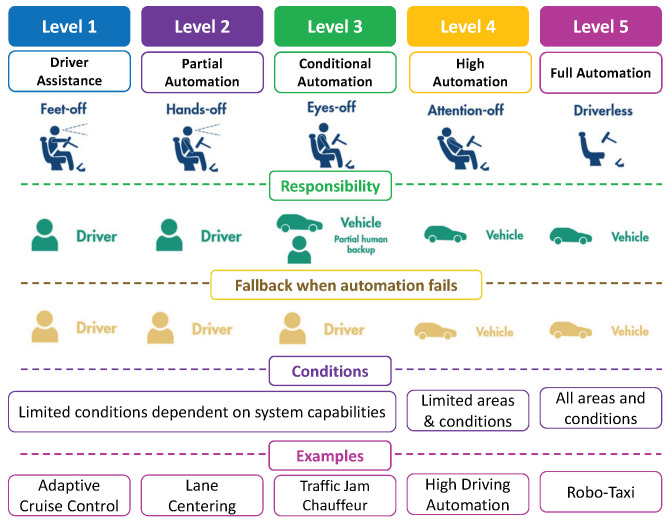
SAE Levels of Driving Automation.

The advancement in sensor technologies, mobile network technologies, and artificial intelligence (AI) has pushed the boundaries of different verticals i.e., healthcare and autonomous driving (AD). Statistics show that more than one million people are killed in traffic accidents yearly, where the vast majority of the accidents are caused by human negligence [1]. It is envisioned that the development of safe and robust AD systems has the potential to drastically reduce road traffic accidents by shifting the entire responsibility and driving control from humans to vehicles. Therefore, the obvious byproducts of AD are expected to be improved road safety and traffic efficiency. The Society of Automotive Engineers (SAE) [2] outlines six levels of design goals for AD, ranging from Level 0 (L0) to Level 5 (L5). It can be seen in Figure 1, L1 indicates no automation and L5 indicates all automation, however, a fully automated vehicle will not have a driver’s cockpit. The evolution from L1 to L5 vehicles necessitates the implantation of various new features in vehicles.

**Figure 2:**
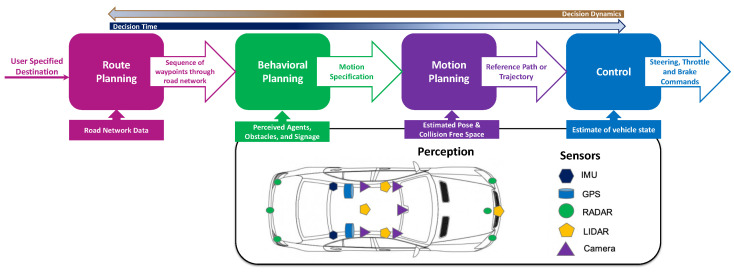
Software Architecture of Autonomous Vehicle.

The core components of an autonomous vehicle (AV) software stack are broadly categorized: into three categories: perception, planning, and control, as shown in Figure 2. Each layer is responsible to carry out the layer’s specific operations, and decisions realizing the inter-layers interactions for different use-case scenarios aiming to achieve a higher level of autonomy in AD. Table 1 presents the space and time horizons required to make the decision at each layer [3]. In what follows next, each component is discussed briefly:

***(i) Perception:*** The perception component of AV collects the information from different on-board sensors (LiDAR, RADAR, and camera) and external sources (high-definition maps), extracts the relevant knowledge, and develop an understanding of the environment focusing on; Which type of objects are in the vicinity of the vehicle? How far is the next obstacle? How effective is the detection of traffic signs, road marking, curves, neighboring vehicles, pedestrians, cyclists, other objects, and so on? The perception has a direct impact on the planning and decision-making of AVs allowing them to react to the events of the environment accordingly. Therefore, the wider and more accurate environment understanding is, the better AVs make the decision [4].

***(ii) Planning:*** Responsibility for making the decisions and providing the AV with a safe and collision-free path towards its destination, taking into account the vehicle dynamics, maneuvering capabilities in the presence of obstacles, along with traffic rules and road restrictions [5].

***(iii) Control:*** Responsibility for translating the AV intentions into actions by taking the generated control signals from the decision-making component and applying them to physical control systems of the AV, for example, steering, throttle, and braking.

An ample amount of research has been carried out toward deploying autonomous vehicles during the last decades. However, still, plenty of issues need to be addressed to achieve higher automation. In connection to this, both industry and research communities have been working on solutions to realize a higher level of AD, therefore, the solution approaches focus on different aspects of AD, i.e., augmenting the vehicle perception, improving the decision-making and control, implanting intelligence in the vehicles, and improving the communication technologies to enable reliable and real-time vehicle-to-everything (V2X) communication. However, the perception systems have been remarkably improved, mainly due to the success of deep learning techniques [6]. The low-level control of the vehicle is already a mature research area and can be solved with classical control theory approaches [7]. However, how to deal with high-level decision-making in a complex, uncertain, and urban environment is a comparatively less explored area [1,8] and forms the main topic of this research.

There are some existing surveys on decision-making for AVs. Schwarting et al. [9] provided a review of some typical methods of decision-making. Katrakazas et al. [5] presented a review of the fusion of solution approaches for planning and decision-making. Li et al. [10] mainly overviewed the research studies conducted on the planning and decision-making technologies at intersections. Leon et al. [11] conducted a review on tracking, prediction and decision-making approaches for AD. Even though these surveys provide a variety of content they did not detail enough on the decision-making approaches. Considering the importance of decision-making technology for AVs and the tendency of the recent development of new approaches, this paper aims to provide a detailed analysis of solution approaches focusing on decision-making for AVs. To equip the readers with the background information on AD and its related concepts, we highly encourage them to look into our comprehensive surveys [12,13] published in reputed journals.

This paper is divided into five sections. Section 2 equips the readers with an overview of decision-making technology. Section 3 provides a comprehensive survey of the research literature on different decision-making techniques and methods. Section 4 outlines the challenges of decision-making approaches and suggests future directions in this area. Finally, Section 5 elucidates the conclusion of the study.

## 2. Decision Making in Autonomous Driving—An Overview

Decision-making is the key player in enabling automated driving and is realized through planning algorithms. The decision-making component is responsible for making suitable driving decisions relying on the context understanding of the environment received from the perception module and then a plan for a drivable path is generated by a path planner which is then passed to the control module for execution hence achieving both safety and traffic efficiency [14].

The planning module of an AV is further broken down into three hierarchical level planners as presented in Figure 2:

***(i) Mission Planning**:* also known as route planner is in charge of finding the optimal global route from a given source to destination taking into account the predefined criteria such as fastest route, shortest route, minimum travel time, and fuel efficiency. The global route is determined once at the beginning of driving by leveraging the map information and occasionally supplemented by real-time traffic information. Therefore, in the case of disturbances and unprecedented events such as accidents or road work modified route planning is required [15].

***(ii) Behavioural Planning:*** the behavioral planner is responsible for decision making ensuring; the vehicle follows road rules and interacts with other agents in a conventional, safe manner while making incremental progress towards the route determined by the mission planner. The behavioral planner is also referred to as tactical decision making or maneuver planning, where the ego vehicle has to deal with a wide variety of traffic situations thus adapting its driving behavior continuously considering the perceived environment. It takes the static, dynamic objects, road blockages, and traffic-free reference into consideration as input and makes the high-level maneuver decisions i.e., when to change lane, merge, turn left, overtakes, or cross interaction and then outputs the decisions to the motion controller to efficiently execute the operations of AV [4].

***(iii) Motion Planning**:* after making the decisions, the motion planner, also known as the local planner, plans a set of actions, a suitable collision-free path, and a detailed trajectory for some period of future time allowing AV to reach its higher-level goals while avoiding the collision.

Within the planning hierarchy, the task of behavioral decision-making is pivotal in translating thoughts into actions. It is a crucial component where intelligent decision-making algorithms are, which make AD genuinely feasible, safe, and fast. Moreover, the functions lying under the scope of behavioral planning define mostly the objectives of higher automation levels. Therefore, this survey focuses on the behavioral planning of autonomous driving.

## 3. The Analyses of Decision-Making Relevant Solutions for Autonomous Driving

**Figure 3:**
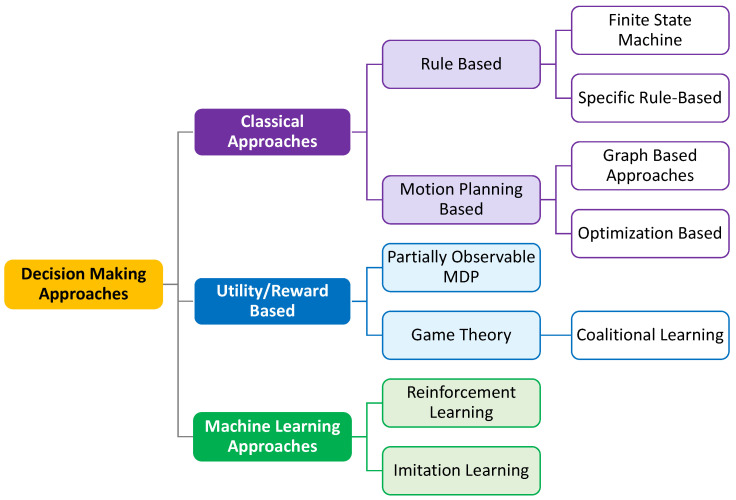
Categorization of Decision-Making Approaches for Autonomous Vehicles.

The behavioral decision-making approaches for autonomous vehicles roughly fall into three main directions: classical approaches, utility/reward-based approaches, and machine learning approaches as shown in Figure 3.

### 3.1. Classical Approaches

The classical methods of decision-making for AD can be grouped into two categories: rule-based methods and motion planning methods. The analysis of these methods is discussed in this section and the comparison of these approaches is presented in Table 2.

#### 3.1.1. Rule-Based Approaches

**Figure 4:**
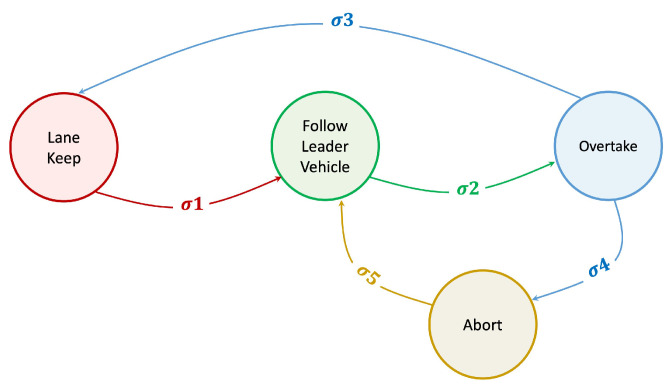
Simple Finite State Machine for Autonomous Vehicle Overtaking Maneuver.

The common representative of this approach is the Finite State Machine (FSM) method. A sample of FSM shown in Figure 4 is a mathematical model with discrete inputs and outputs where the actions are generated to react to the external events resulting in transitioning the states of the agents to another state. Based on the logical structure of the states, the FSM models are categorized into three types: tandem type, parallel type, and hybrid type, which have been widely implemented in autonomous vehicles [16].

The winning team, Tartan Racing, of the DARPA Urban Challenge, implemented the behavior generation component leveraging the hierarchical FSMs [17]. They broke down the mission task into high-level behaviors such as drive-down road, handle intersection, and achieve-zone-pose, with their simpler sub-behaviors aimed at accomplishing the mission planning task. The state machines triggered the different behaviors based on the progress reported by the motion planners and the objectives set out by the mission planner [18]. Similarly, another team securing second place in the DARPA challenge implemented the behavioral planner utilizing a hierarchical FSM with a total of thirteen states categorized into normal behavior and exception states to execute the tactical decision such as changing lanes, merging, and avoiding obstacles [19].

Wang et al. [20] employed the hierarchical FSM to develop the three-layered FSMs decision-making framework for the vehicle behavior of AV. The top layer was responsible to classify the scenario considering the relative position of the ego vehicle and the information of its neighboring vehicles, and the middle layer determined the optimal driving behavior by evaluating the optimal energy efficiency value of the potential vehicle behavior targeted at multiple criteria factors such as driving efficiency, safety, and the grid-based lane vacancy rate, lastly, the lower layer generated the state transition matrix combined with the results of the middle layer to decide the optimal driving strategy for AV to pass way in the region. The simulation-based results concluded that the proposed framework could choose the optimal driving decision in complex road scenes while considering safety and traffic efficiency.

Similarly, in another study [21], the FSM separately implemented two longitudinal and four lateral state transitions contributing to an emergency stop assistant on highways. Unlike the rule-based approaches, the FSMs could directly execute the pre-planned sequence of actions and states which were then mapped with path generation and control. The FSMs are also considered, as state classifier algorithms, thus making them a simple communication framework for collective and driver-shared driving [3]. Ziegler et al. [22] developed multiple statecharts in parallel to handle the concurrent states machines which were good at executing simultaneous actions in a decision process such as yielding and merging maneuvers. To conquer the limitations of classical FSM, a double-layer FSM was implemented to minimize the complexity of driving behavior transfer. It utilized a rule-based decision approach to plan maneuvers such as structured driving, lane changing, turning, and so on aimed to enhance stability under different driving scenarios [23].

Concludingly, some of the limitations of FSM-based studies are that they are fully reliant on knowledge certainty and cannot be generalized to unknown scenarios. They are unable to manage large complex networks, resulting in state and transition explosions. To have a completely reactive system; each state must be able to transit to every other state making it a fully connected graph. Therefore, the modification of FSM is labor-intensive making them difficult; to collaborate as they expand [24].

Researchers also focused on implementing the decision-making approaches using a specific rule base for a particular scenario. Rule-based decision-making approaches make use of a rule database built on combining the traffic rules and laws, driving experiences, and driving knowledge to make the decisions. Some of the other rules for decision-making could be speed limits, work zone, stopping at the stop sign and intersection precedence handling [25]. The algorithms of this approach make decisions by considering the states of neighboring vehicles without taking their dynamics into account, hence predicting their trajectories. Therefore, these algorithms are deemed applicable to the scenarios and task-driven autonomous driving modes.

Antonio et al. implemented a decision-making framework without utilizing a detailed priori map [26,27]. A maneuver planner was developed, with four different planning modes to address all possible situations of the environment when analyzing the predicted motion of nearby objects together with the current planned trajectory of the ego-vehicle: (i) re-plan from the current pose; (ii) extend the current trajectory; (iii) avoid static obstacle and (iv) avoid the dynamic obstacle. Finally, a hybrid flow diagram was designed to make maneuvering decisions based on a series of rules/conditions of the surrounding environment to generate the final trajectory of the ego vehicle. Chang et al. [28] designed a multi-point decision-making framework combining real human driving data and vehicle dynamics for human-like autonomous driving. To control the vehicle steering, the framework determined the optimal turnaround maneuver to reduce the time required to execute the task based on a rule of minimum road width to perform a U-turn. Behavioral planning and decision-making are some of the biggest challenges for highly automated systems. Therefore, to overcome these challenges, Piotr et al. [29] contributed with a framework combining a rule-based and hierarchical behavior-based architecture for tactical and strategical behavior generation in automated driving. The results showed that the proposed framework has a high degree of generality and expandability, and can be combined with several scenarios.

Humans can recognize the maneuver intentions of neighboring vehicles by observing lateral and longitudinal motion cues. Therefore, to adapt this ability to the AVs, Nilsson et al. [30] implemented simple logical rules to recognize the intentions of highway maneuvers. The experimental results showed that the proposed framework could correctly recognize the intentions of left and right lane change maneuvers with an accuracy of 89% approximately. Aksjonov et al. [31] also proposed a rule-based decision-making system for AVs aimed at solving the challenge of complex intersections with mixed traffic environments. The system was built to prioritize road safety and avoid collisions with other road users at any cost. The proposed framework relied on the on-vehicle perception and localization sensors allowing the AV to operate alongside the human-driven vehicle and without vehicle-to-vehicle (V2V) communication technology.

To sum up, the decision-making process in L2 to L4 autonomous driving is mainly driven; by rule-based methods implemented as handcrafted state machines and rules. These methods are simple, reliable, highly interpretable, provide a breadth of decision-making, and are straightforward to implement. In simple scenes, their applicability is superior compared to other feasible methods. However, some of the limitations of these approaches are that they do not deal with the input uncertainties and cannot generalize to unknown situations, which makes them difficult to scale them to the complexity of real-world driving where the events may be unprecedented and unknown. Furthermore, when dealing with complex urban environments, such as road intersections, where various uncertainties exist, a rule-based approach cannot maintain safe and efficient driving [10,15,32,33,34].

#### 3.1.2. Planning-Based Methods

Another group of algorithms addresses the decision-making task as a motion planning problem. Commonly, a prediction model is developed to predict the motion and intention of the surrounding vehicles, and then the behavior of the ego vehicle is planned accordingly. This results in reactive behavior since the predictions are independent of the ego vehicle plan. Therefore, the interaction between the ego vehicle and other agents is not explicitly considered [8,35]. The current states such as the position, velocity, and yaw angle of the nearby vehicles can be measured using a perception module which is then utilized to make predictions for each time step over the total horizon. Two types of motion-planning algorithms for decision-making are discussed briefly in this subsection.

##### Graph-Based Search

The graph-based method is often used in mobile robotics and can assist AVs in finding a path within the free space. Commonly, a tree-like graph is used to model different decisions and their consequences in choosing the optimal action. Hubmann et al. [36] introduced a simple rule-based classifier to model the road users and then, the A* graph search method was utilized with invisible collision states as a heuristic. This method was tested on the road as well as in BMW’s Highly Automated Driving Framework simulator. Another graph-based approach is using Rapidly Explored Random Trees (RRTs). In another study, Liu et al. [37] leveraged the cooperative perception to execute the motion planning via an RRT-based framework. The map was first divided into a grid using the occupancy grid map, and then a cost map was generated to keep the vehicle in the middle of its lane on the road, and planning was then handled by the Anytime RRT algorithm. Arab et al. [38], used the Sparse Stable Trees with the RRT* approach for motion planning to simplify the problem by pruning non-useful nodes and model predictive control. Li et al. [39] combined the sampling and graph searching-based methods in the Frenet frame and simplified the trajectory searching space to improve efficiency. In some of the recent studies, Heged et al. [40] and Speidel et al. [41] also implemented the graph-based motion planning algorithms for AVs. Furthermore, readers are encouraged to look into [40] for a detailed discussion on graph-based approaches.

##### Optimization-Based Models

Another approach to solving the motion planning problem is to use optimization-based techniques. An optimization method such as Model Predictive Control (MPC) is an effective method and has been broadly used for planning and decision-making problems for AVs. In MPC, motion planning is considered an optimization problem where the system’s dynamics are used as constraints alongside obstacle avoidance requirements. This optimization problem is commonly solved over a finite time horizon for real-time tractability, and re-planning is frequently used to account for uncertainty and updated information.

The automatic merging maneuver is one of the most challenging maneuvers since it must be completed in a dynamic traffic environment within a limited distance. Li et al. [42] proposed an integrated path planning and trajectory tracking algorithm based on MPC to execute automatic lane merge in a mixed traffic environment such as with manual vehicles, semi-AVs, and fully AVs. A comparison of the simulation-based results was made between the proposed algorithm and a benchmarked two-layer control strategy. Overtaking is another complex maneuver. The available solution approaches to autonomous overtaking are limited to simple and static scenarios. Palatti et al. [16] developed a framework for the behavior and trajectory planning for safe autonomous overtaking. A simple rule-based finite state machine was used; to determine the optimal maneuver at each time and a combination of safe and reachable sets was used to iteratively build intermediate reference targets based on the current maneuver. In addition, the proposed method had a novel feature that allowed the AV to abort the overtaking and merge back into the lane if safety was compromised. The feasible and collision-free trajectories were planned using the nonlinear MPC. Following this, Lin et al. [43] also implemented a safe overtaking maneuver for an AV based on time-to-lane crossing estimation and the MPC scheme. Viana et al. [44] also proposed the non-linear MPC to solve the problem of cooperative optimal trajectory generation for AD. The simulation-based results in CarSim showed the effectiveness of the proposed model for a cooperative overtaking scenario.

Bey et al. [45], implemented the MPC to regulate the whole traffic situation, where high-level behaviors of an ego vehicle were generated, and other vehicles were indirectly influenced by the ego’s behaviors. Yang et al. [46], modeled the vehicle-pedestrian interaction through a multi-state forced pedestrian motion prediction model, and then MPC was used to generate low-level control commands for the ego vehicle. Sun et al. [47], implemented MPC combined with inverse reinforcement learning (IRL) to develop a more suitable cost function. In another study by Werling et al. [48] the optimal control method was applied to highway driving scenarios.

### 3.2. Reward/Utility Based Approaches

Another category of solution approaches relies on a reward or utility function to make decisions. This section discusses two utility-based decision-making approaches for autonomous driving.

#### 3.2.1. Partially Observable Markov Decision Process

The partially observable Markov decision process (POMDPs) extends the Markov Decision Processes (MDPs) to situations in which the other agents’ intentions and re-planning strategies are encoded in hidden variables and cannot be directly observed. Their capability to cope with probabilistic uncertainty of the observed environment makes them an essential topic in automated driving. Usually, these models have access to noisy or imperfect observations of the state, and the agent then acts in response to an estimate of the true state of the world. Given a set of possible states, actions, reward functions, conditional transition probabilities, and observations, the POMDP allows an agent to decide the best course of action to take. The agent’s objective is to evaluate every action sequence and consider its impacts to optimize the overall long-term predicted reward over a period of time.

The POMDP is a mathematical framework to make decisions in the presence of uncertainty, sensor noise, and other constraints. It is capable of simulating uncertainty in both the current state and uncertainty in the future evolution of the traffic scene, therefore, modeling interactive behavior can be divided into two classes: offline and online methods. The offline techniques often concentrate on calculating the optimum course of action for every possible belief state, rather than only the current belief state. As a result, they establish a policy before the execution specifying the best course of action to take in every possible circumstance. On the other hand, online methods are more adaptable than offline methods since a policy is determined as the approach is being executed. However, the restricted number of computational resources available necessitates careful problem formulation and lowers the quality of the solutions.

Liu et al. [54] proposed the intention prediction approach for AVs utilizing the hidden Markov model (HMM) to accurately forecast the upcoming driving intents of human-driven vehicles in a mixed-traffic environment. HMMs with various driving intentions were trained and tested on the data collected from a flyover. The experimental outcomes demonstrated that the suggested framework performed better at predicting driving intentions than logistic regression. A human-like decision model for unsignalized intersections was proposed by Hsu et al. [55] by leveraging the intention-aware method to forecast the intentions of other drivers and the movement of obstacles based on a convolution neural network (CNN) with multiple objects tracking and Kalman-Filter operations. Another study by Song et al. [56] implemented an intention-aware decision-making algorithm for an urban environment comprised of an uncontrolled intersection. To predict the low-level interaction intentions as well as the high-level motion intentions, a continuous HMM was developed and then a POMDF framework was modeled to design the AD decision-making system. The distance to the intersection, longitudinal velocity/acceleration, and yaw rate were used as inputs to anticipate drivers’ lateral and longitudinal intentions at an intersection with a deterministic setting and resultantly they got a reasonable prediction performance. Hubmann et al. [57] implemented the online POMDP with the intended routes of the neighboring vehicles as hidden variables, with a particular emphasis on the scenario where the vehicles merge due to a reduction in the number of lanes on city roads and taking into account the interaction between drivers.

**Figure 5:**
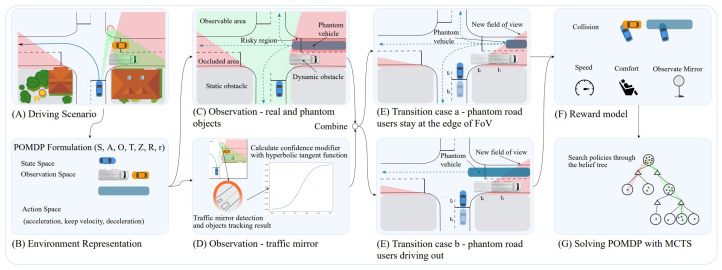
A framework of traffic mirror-aware POMDP behavior planner.

Huang et al. [58] developed an IMM-based POMDP decision approach for a mandatory lane change maneuver leveraging collision-risk function incorporating the time-to-collision, inter-vehicular time, and the collision function. The suggested collision-risk function was composed of two components: the vehicle impact factor and the collision function, which calculated the likelihood that the AV would collide with nearby vehicles. The authors concluded that given the collision-risk function and the probability distribution of the statuses of nearby cars in the future, the suggested POMDP decision-making algorithm could detect whether the AV accelerated, or decelerated lane shifting and obtained the acceleration corresponding to each path point. Coskun et al. [59] suggested a lane-changing model made up of two components: a threat assessment that makes use of fuzzy logic to evaluate how traffic players interact and a decision-making method based on the MDP concept. In another study, Song et al. [60] developed a POMDP model for decision-making for lane-changing and lane-keeping operations. To represent the candidate policies utilizing path and velocity profiles throughout the policy generation process, a maneuver-based decomposition method was created. Then, a deterministic machine learning (ML) model was deployed to identify the driving intents of human-driven vehicles. Lastly, a situation prediction model was put forth to calculate potential future actions of other vehicles considering cooperative driving behaviors. To enhance the AV’s capability to handle a variety of occluded driving circumstances, Zhang et al. [61] presented an interesting behavior planner incorporating the traffic mirror awareness based on the POMDP model, see Figure 5. The proposed approach created phantom traffic participants in hazardous occluded locations and predicted the likelihood of their existence using contextual data and uncertain traffic mirror detections. The experimental results demonstrated that in the presence of obstructions at crossings and crosswalks, the planner drove more safely and effectively by utilizing traffic mirror detection. However, the lack of a V2X module, which could have provided measurements of dynamic road users with uncertainty in unobservable locations, is one of the main shortcomings of the proposed approach.

Overall, the POMDP approach is extremely advantageous, allowing all sources of uncertainty to be modeled but real-time implementation is usually a problem due to the computational complexity [49]. Some of the main limitations of the POMDP methods are they suffer from the curse of history, their computational complexity growing exponentially with the planning horizon, and the curse of dimensionality. The size of the state space grows exponentially with the number of state variables. Therefore, the POMDPs need to plan on a (|*S*| − 1) dimensional continuous belief space [62]. The limited available computational resource for online methods requires careful problem formulation. However, the main drawback of the offline methods is that they are designed for specific scenarios. Hence due to the wide variety of real-world scenarios, it becomes impossible to pre-calculate a policy that is generally valid for all scenarios. Computing approximate offline solutions, even for extremely simple POMDP problems, may take several minutes to many hours. On the other hand, decisions must be updated frequently (e.g., every 100 ms) while making decisions in traffic scenarios Besides, solving the most general POMDP is intractable in real-time applications [9].

#### 3.2.2. Coalitional Learning Approaches

Game theory (GT), is an analytical framework with several mathematical tools used to study the cooperation, conflict, and complicated interactions between numerous independent rational players/agents. The players are also referred to as decision-makers because one player’s decision may have an impact on the other players. GT has had a revolutionary impact on a wide range of disciplines over the past few decades, including economics, politics, communication, wireless networks, computer science, and more. The game-theoretical framework has two primary branches: non-cooperative game theory (NCGT) and cooperative game theory (CGT). The primary focus of NCGT is on analyzing and modeling the competitive behavior of players and the strategic decisions that emerge from interactions between these rival players. Each player independently determines its strategy with the goal of either enhancing performance or minimizing losses. As a result, NCGT cannot support binding agreements. While the CGT simulates cooperative behavior and agreements to distribute cooperative gains among players. CGT is further classified into two approaches; Nash bargaining and coalitional game theory [63].

Although a few branches of game theory (GT) such as cooperative, leader-follower games [64], etc are fitting approaches, we focus on the analysis of coalitional games in the context of behavioral decision-making in this paper for the following reasons:

1. Coalitional GT for autonomous driving is a relatively newer and less explored area.
2. It provides very fitting characteristics for realizing the solutions of complex and urban driving, specifically for short-term, highly dynamic, and L4/L5 platooning.
3. The approaches such as cooperative GT for collaboration are researched extensively and publications may be found e.g., [65,66,67,68].

Coalitional GT has been employed in a number of applications and has proven to be a potent framework for developing reliable, usable, and effective cooperation strategies in a variety of settings. It primarily deals with forming cooperative groups referred to as coalitions, allowing the cooperating players to strengthen their position in a game. The coalitional game is made up of two components: (i) the set of players which interact with each other to form cooperative coalitions and make agreements among them to act as a single entity in the given game; (ii) the coalition value which denotes the utility of the coalition in the game. The coalitional game may be either a TU game (transferable utility) or an NTU game (non-transferable utility). In TU games, the utility of the coalition is a real value that can be distributed among the coalition’s members in any way. The coalition value in NTU games, however, is not a real number but rather a vector payoff, where each element denotes the potential reward payoff for each coalition member [69]. Mathematically the coalition game can be defined as below:

**Definition 1** (Coalition Game)**.** *A coalitional game is defined by a pair 〈N,ν〉, where N={1,2,…,n} is the finite set of players who seek to form coalition S such that $S\in 2^{N}$. The S consisting of only one player is referred to as a single-player coalition and S with all players is referred to as a grand coalition. The ν is a real-valued function, called characteristic function such that $\nu :2^{N}\to R$ maps each possible coalition S⊆N to its payoff ν(S).*

**Figure 6:**
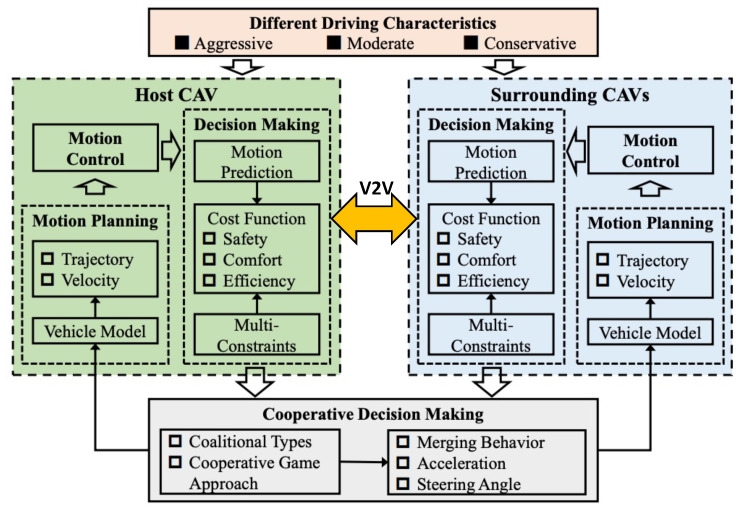
Coalitional game theory based decision-making framework for CAVs.

**Figure 7:**
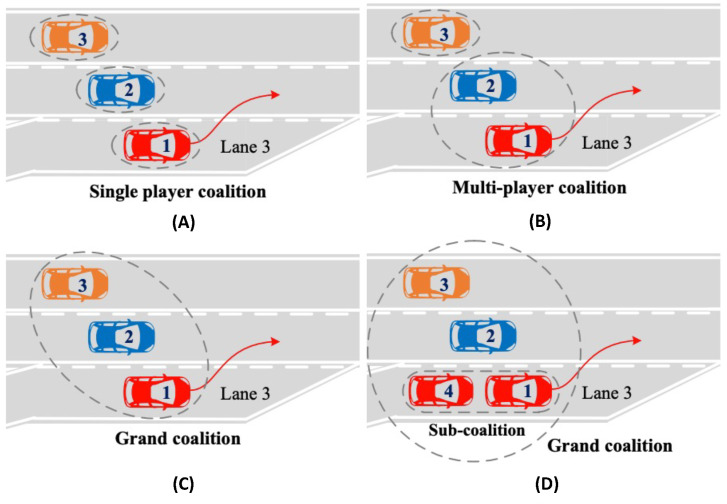
Four types of coalitions. (**A**) The single player coalition; (**B**) The multi-player coalition; (**C**) The grand coalition; (**D**) The grand coalition with a sub-coalition.

In what follows next, the relevant literature review of coalitional game theory is discussed below:

In highway settings, one of the causes of traffic congestion is vehicle merging. Several factors, including traffic dynamics, driver preferences, and travel aims, make it difficult for vehicles to perform merging moves. To address the multi-lane merging issue for CAVs, Hang et al. [65] developed a cooperative decision-making framework based on coalitional GT, see Figure 6. The motion prediction module was first developed to forecast the motion states of the vehicles. The cost function for decision-making was created taking into account certain constraints including safety, comfort, and traffic efficiency based on the estimated motion states of the ego and surrounding vehicles. After creating the cost function, the coalitional game was used with MPC to manage the coordination and decision-making of CAVs at a multi-lane merging zone. Finally, the experimental findings on two case studies, with four types of coalitions (see Figure 7), and various driving characteristics demonstrated that CAVs were capable of making sane decisions. Additionally, the cost value of each CAV in the grand coalition was lower than that in the coalition made up of a single player, demonstrating the superiority of the grand coalition. In another study, Hang et al. [70] built the cooperative lane-change decision-making framework for AVs based on a cooperative coalitional game strategy that took into account human-like driving traits like aggressive, moderative, and conservative. Three performance indices—safety, comfort, and efficiency were used to build the cost function for making decisions. Additionally, the cooperative coalitional game approach was used to transform the cooperative lane-change decision-making problem into an optimization problem with multiple constraints.

It can be difficult to manage traffic at junctions, especially in cities where it tends to get much worse. Hang et al. [71] addressed the coordination and decision-making problem of CAVs at the unsignalized intersection. First, a Gaussian potential field approach was employed to construct the driving risk assessment algorithm, which reduced the complexity of the decision-making system by evaluating the safety risk of nearby vehicles. The driving safety and passing effectiveness of the CAVs were taken into account to build the decision-making cost function. Following that, the authors created several decision-making constraints, including control, comfort, efficiency, and stability. Finally, based on the cost function and constraints, two types of fuzzy coalitional game techniques—(i) single-player coalition and (ii) grand coalition were developed to address the decision-making problem of CAVs at unsignalized junctions representing both individual and social advantages. The experimental results showed that the suggested decision-making framework might assist CAVs with safe, effective, and rational decisions. Hoai et al. [72] implemented a coalitional game-based approach among intersection controller agents to reduce the amount of time that cars are forced to wait at various intersections. This method relies on real-time traffic flow data collected from CAVs, which then control traffic at the intersections. To demonstrate the success of the suggested technique in terms of the number of vehicles through intersections at a given time and the waiting time of vehicles, different rates of traffic flow were developed. To simulate the traffic light control at intersections, Netlogo, an agent-based modeling simulator, was used. Results from simulations indicated that the proposed strategy, when compared to the conventional approaches, greatly outperformed them in terms of controlling the traffic at various crossings. In another study, Wei et al. [73] implemented a hierarchical game-in-game structure to improve traffic safety by lowering collision rates while also enhancing intersection throughput. A connected, multi-layered strategy that took into account coalitional and non-cooperative games was formulated. To increase throughput and the smoothness of traffic moving through the intersection, a coalitional game that groups vehicles into platoons and schedules their passage through the intersection was proposed in the first layer. To prevent collisions inside the intersection, a strategic game was developed for the second layer. Finally, the proposed game-in-game framework found the intersection traffic management equilibrium solutions.

To maximize road safety, traffic flow, fuel consumption, and platoon stability, Angel et al. [74] used the coalitional GT to establish the vehicle platoon and at the same time managed the intra- and inter-platoon coordination utilizing a cooperative communication strategy. Three utility functions; individual, coalitional, and global functions were used to examine the game. The local environment, including its state vector and the information supplied by the infrastructure, was considered an individual utility. The coalitional utility motivated the formation of a coalition, followed by the global utility, where some of the players were not individuals but rather formed coalitions. The proposed theoretical framework is then validated on two parameters—load per path and transit time to destination. Similarly, in another research, Khan et al. [75] used a hardware wireless Convoy Driving Device (CDD) to develop a coalition formation strategy to help the driver decide whether to join or leave a platoon, influencing the speed and formation of the platoon. The CDDs could communicate with one another and make decisions about the formation of a platoon based on a variety of parameters, including the vehicles’ current speed, the desired speed, vehicles’ limitations, and the speed limits on the roads. The vehicles negotiated and agreed on a similar platoon speed and then changed their speed to keep the platoon steady, ideally with uniform following distances. Besides, two algorithms were developed to determine whether to join the coalition. The experimental results showed that rational coalitions can be created by utilizing the speed and proximity information of neighboring vehicles. The social potential fields-based influence scheme, however, can be used not only to make coalitions but also to regulate the V2V distance within the coalition. The motion controller and spacing policies have an impact on the platoon’s overall performance and profit. A systematic spacing policy was presented by Liu et al. [76] to study the spacing decision based on coalitional GT. Based on the concepts of bionic motion, a flock’s model was employed as the payoff function. To allocate the spacing fairly, the characteristics function for the platoon was developed based on the Shapley value and average lexicographic. The outcomes demonstrated that the suggested method improved both the convergence of longitudinal following error and the steady time for consistency control.

Hadded et al. [77] implemented a shared transportation system use case in an urban setting where human drivers were contracted to pick up abandoned vehicles. Each driver, termed a platoon leader, was responsible for driving gathered vehicles as a platoon to return them to a specific location, like an airport or a train station. To address this issue, a hedonic coalition game was constructed to determine the following: (i) the distribution of unused vehicles to the least number of platoons; (ii) the optimum tour for each platoon, and (iii) the minimal energy required to collect all of these vehicles. In the coalitional GT, the authors considered the parked vehicles as the players and the coalitions as the vehicle platoons. Three optimization criteria were used to assess the solution’s quality after the game converged to a stable result. The simulation-based results demonstrated that the suggested technique was effective at resolving the multi-objective optimization problem.

By reviewing the literature we summarize some of the main limitations of game theory as below:

1. In every theoretical framework, modeling a system necessarily entails some amount of abstraction. On the other hand, game theory has a particularly high level of abstraction since there are so many implicit assumptions that must be taken into consideration in game-theoretic models [78].
2. Microscopic driving decisions based on the application of game theory modeling could result in computationally slow methods, making the chosen approach unsuitable for real-time simulation. This issue becomes even more obvious when working with more intricate driving scenarios [79].
3. Increasing system complexity necessitates the use of greater computation resources and more potent decision-making execution capabilities [80].

Furthermore, the comparison of coalitional game theory studies based on different parameters is given in Table 3.

### 3.3. Machine Learning Approaches

Machine learning (ML) has recently attracted attention in the field of autonomous driving due to advances in deep learning. The advantage of this approach is that it does not rely on hand-crafted rules and scales well with data, improving performance as more data is utilized for training. Consequently, this method has a great deal of potential to manage a wide range of driving scenarios. A plethora of ML approaches have been investigated and are contributed by the research community for different use cases enabling a higher level of autonomous driving. For a ready reference, readers are directed to some recent surveys [81,82,83] for a detailed discussion. However, this section analyzes the two most popular ML paradigms for planning and decision-making in AD.

#### 3.3.1. Reinforcement Learning

**Figure 8:**
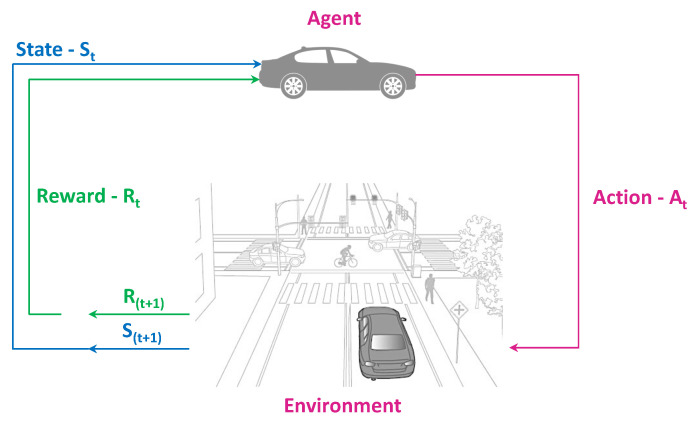
Illustration of Reinforcement Learning Approach.

Reinforcement Learning (RL) is a branch of machine learning that deals with the issue of automatic learning and the best choices over time. RL enables an intelligent agent to learn from its mistakes and experiences. The RL agent acts in the environment to receive rewards where the goal of the agent is to choose the action that will maximize the expected cumulative reward over time. An RL agent can be characterized as an MDP Process in the following way: the agent interacts with the environment by taking actions and getting feedback and rewards. As shown in Figure 8 at each time step *t* the agent obtains a representation of the environment state $s_{t}\in S$. Based on this state the agent decides on a course of action $a_{t}\in A$. Any action is chosen depending on the agent’s behavior, often known as the policy which instructs the agent on the best course of action for every potential state. As a result of each action, the agent is rewarded with a reward $r_{t}\in R$ and observes the next state $s_{t+1}\in S$. The process of receiving a reward can be described as an arbitrary function, *f*. At each time *t*, we have:

$$f\left(s_{t},a_{t}\right)=r_{t}$$

RL plays an imperative role in the decision-making process of AD, which enables AVs to acquire an ideal driving strategy through constant interaction with the environment. Lv et al. [84] developed a deep RL (DRL)-based motion planning technique for AD scenarios including an AV merging into two-lane road traffic flow and executing lane-changing maneuvers on highways. An improved version of the DRL algorithm based on a deep deterministic policy gradient (DDPG) is created with well-defined reward functions. Specifically, safety rules, a safety prediction module, trauma memory, and a dynamic potential-based reward shaping function are adopted and implemented to further improve safety and hasten the learning of lane-changing behavior. To identify a risk-aware driving decision approach with the lowest risk for AD, Li et al. [85] suggested a lane change decision-making framework based on DRL. Firstly, a probabilistic model-based risk assessment method was presented to evaluate the driving risk utilizing position uncertainty and distance-based safety KPIs. Then, a risk-aware decision-making algorithm was designed to use DRL to identify a strategy with the lowest expected risk. The proposed solutions were then tested in two scenarios using the CARLA simulator. The findings demonstrated that the suggested methods can produce safe driving strategies and provide better driving performances than classical techniques.

Roundabout driving can be challenging for both manual and AVs. Cuenca et al. [86] presented a method based on the Q-learning algorithm to train an AV agent how to safely navigate on roundabouts. The CARLA simulator is used to implement the suggested learning algorithm. The algorithm is trained through a number of simulations in two situations: navigating a roundabout with and without surrounding traffic. The outcomes demonstrated that the Q-learning algorithm-based vehicle agent can learn effective and smooth driving techniques to execute maneuvers at roundabouts. In another research, an optimization embedded reinforcement learning (OERL) approach is proposed by Zhang et al. [87] to achieve adaptive decision-making under the roundabout. The promotion is the modified actor of the Actor-Critic framework, which embedded the model-based optimization method in RL to explore continuous behaviors in action space directly. As a result, with high sample efficiency, the proposed method may concurrently identify the appropriate acceleration and action time at the medium-scale and macroscale levels (whether to change lanes or not). Prathiba et al. [88] proposed a hybrid DRL and genetic algorithm (DRG-SP) for smart platooning of AVs. By leveraging the DRL technique, the computational complexity is addressed, and the highly dynamic platoon scenarios are supported. Adopting the Genetic Algorithm in DRL solved the slow convergence issue and provided long-term performance. The simulation’s findings showed that Smart-Platooning efficiently creates and maintains platoons by reducing traffic jams and fuel consumption.

Lack of interpretability is one issue with learning-based approaches for autonomous driving. The learned deep neural network policy is similar to a black box which is not desirable since AD is a safety-critical application. It is crucial to understand how the autonomous vehicle understands its surroundings. The existing end-to-end approaches frequently lack interpretability and can only handle basic driving tasks like lane keeping. An interpretable DRL strategy for end-to-end autonomous driving capable of handling challenging urban scenarios was proposed by Chen et al. [89]. They introduced the sequential latent environment model and learned it jointly with the RL process. With the help of this latent model, a semantic bird-eye mask can be created, which is required to connect with some intermediate attributes in the current modularized framework to explain the actions of the learned policy. The sample complexity of RL is also greatly reduced by the latent space. Comparative experiments in a realistic driving simulator revealed that the proposed technique outperformed numerous baselines, including DQN, DDPG, TD3, and SAC in urban settings with congested surrounding vehicles. In another research, Wang et al. [90] proposed a latent space RL approach for interpretable decision-making for AVs at highway on-ramps. The proposed approach is based on the latent model, a combination of the hidden Markov model, and a Gaussian mixture regression model (HMM-GMR). Due to the high-dimensional input and lack of task understanding, the traditional decision-making approach has difficulty comprehending the environment. HMM-GMR model can be used to acquire the interpretable which provides semantic information and environment knowledge. The latent model is used to minimize the dimension of the interpretable state by extracting underlying task-relevant data in a framework that unifies representation learning with the DRL technique. The results of the experiments are provided, and they demonstrated the ideal balance between driving safety and efficiency in the complex situations of merging highway on-ramps. Chen et al. [91] put forth a novel end-to-end technique for AD perception. Sequential latent representation learning is used to introduce a latent space that contains all pertinent information useful for perception. With only minimal human engineering efforts and without maintaining any maps online, the learned end-to-end perception model was capable of solving the detection, tracking, localization, and mapping challenges all at once.

Some main drawbacks of RL techniques are (i) the policy learned by RL algorithms is through trial-and-error, which is wholly reliant on the experience gained through interactions with environments; (ii) poor stability and over-fitting in DRL methods; (iii) the optimal policy learning problem is notoriously difficult when it comes to large amounts of acquired data referred to as the sample efficiency problem [92]; (iv) performance greatly depends on how the reward function is designed; (v) challenging to design the reward function for complex tasks, which has a significant impact on policy performance. In addition, readers are encouraged to look into some relevant surveys on reinforcement learning approaches in AD [93,94] to deep dive into these approaches.

#### 3.3.2. Imitation Learning

Imitation Learning (IL) is a supervised learning method that uses datasets of expert demonstrations (usually made by humans) to train a system to emulate the given expert in a variety of autonomous driving scenarios. Alternative deep learning (DL) techniques, such as deep reinforcement learning techniques, have been used to solve the issue. However, these approaches are constrained due to the intricacy and safety-critical nature of the driving task. With IL, DL techniques can be trained to a level that is very close to humans by using readily accessible, easily collected large-scale datasets of human driving. For this reason, IL is the main topic of the majority of the literature. In what follows next, we discuss the research on IL in autonomous driving decision-making.

Chen et al. [95] stated that the current IL approaches for autonomous driving are hardly up to the task in complex uncertain urban environments. Furthermore, using a deep neural network strategy does not ensure safety, either. As a result, they developed and implemented a system to learn a driving strategy in general urban scenarios using offline recorded expert driving data, which also improved the safety of collision avoidance. The proposed system was tested using the CARLA simulator, and the experimental results indicated that the framework is capable of obtaining a deep convolutional neural network policy that is wise enough to achieve high performance in simple urban driving scenarios. Controlling a vehicle when it enters a roundabout in an urban environment is a challenging task, even for a human driver. To address this issue, Wang et al. [96] suggested a unique imitation learning-based decision-making framework to offer suggestions for joining roundabouts. The proposed approach used deep policy networks to make decisions about the ideal time to enter a roundabout using observations from a monocular camera mounted on a moving vehicle as input. The domain expert-directed learning framework can not only facilitate better decision-making but also hasten the convergence of deep policy networks. To evaluate the performance of the proposed approach it is compared with the state-of-the-art supervised learning techniques, including support vector machines, k-nearest neighbor, and DL-based approaches. The experimental findings showed that the imitation learning-based framework for making decisions worked better than supervised learning techniques and can be used in a driving assistance system enabling better decisions when approaching roundabouts.

Adversarial Inverse Reinforcement Learning (AIRL) is one of the most advanced imitation learning techniques that can concurrently learn a behavioral policy and a reward function, however, it has only been demonstrated to work in static, unchanging situations. Wang et al. [20] enhanced and stabilized AIRL’s performance by supplementing semantic rewards to the learning framework. Moreover, they modified the enhanced AIRL to a more demanding decision-making task in an environment that is highly interactive for autonomous driving. In another study Yun et al. [97] presented a randomized adversarial imitation learning (RAIL) algorithm. The RAIL is a new imitation learning technique that does not use any derivatives and is designed to mimic the coordination of several advanced driver assistance systems (ADAS) functions while driving autonomously. As a result, it mimics the actions of the decision maker who controls the operation of autonomous driving with several ADAS functions. The suggested approach can be used to train the decision-maker who uses LIDAR data and controls autonomous driving in multi-lane complex highway settings. Recent advancements in multi-agent imitation learning have shown encouraging results for simulating the actions of drivers. Nevertheless, it is difficult to record emergent traffic characteristics that can be observed in real-world datasets. Such behaviors result from the numerous local interactions between agents that are frequently overlooked in imitation learning. Bhattacharyya et al. [98] introduced Reward Augmented Imitation Learning (RAIL), which integrated reward augmentation into the multi-agent imitation learning framework and allowed the designer to explicitly express past knowledge systematically. The authors demonstrated that under the use of reward augmentation, convergence guarantees for the imitation learning process are maintained. The effectiveness of this approach is validated in a driving scenario in which the proposed algorithm is used to learn driving rules that control the entire traffic scene. Additionally, the results showed enhanced performance compared to conventional imitation learning methods for both the local behaviors of a single agent and the behavior of emergent characteristics in complicated multi-agent environments.

By reviewing the literature carefully, we highlight some of the main limitations of IL approaches (i) data-hungry deep learning technique and its performance is limited to the level of the expert policy; (ii) expensive or even impossible to obtain supervised data in some circumstances; (iii) due to the real-time nature of many applications, the learning algorithms are constrained by computing power and memory limitations, particularly in robotic applications that require onboard computation to perform the real-time processing [99]; (iv) traditional IL methods suffer significantly from learning hierarchical policies when the imitative agent encounters an unobserved state by the expert agent [100]; (v) the policy never outperforms the suboptimal expert performance, and the effectiveness of IL is still heavily dependent on the expert policy’s quality. Furthermore, for a ready reference, we also direct the readers to some recent surveys [101,102] to deep dive into these approaches.

## 4. Challenges and Future Recommendations

This section spotlights the research gap, challenges, and future directions for decision-making and behavioral planning.

### 4.1. Explainability in Decision Making

The planning component is a crucial aspect of AVs as they execute sophisticated maneuvers in dynamic, complex, less structured, and cluttered environments such as urban roads, and town centers with a lot of pedestrians, cyclists, and other road participants. Furthermore, the traffic elements such as roadside infrastructures, road networks, road signs, and road quality are dynamic and can change with time; this makes AVs frequently update their plans as they operate. A stakeholder riding in an AV might therefore get confused if the AV modifies its trajectory or execute a maneuver without providing an explanation. The adoption of increasingly sophisticated techniques does not only have advantages, as they are becoming more and more opaque. The lack of transparency means that certain real-time decisions made by AVs are neither recognizable nor understandable to the user, the developer, nor the legislator. The AI’s opaque behavior is commonly referred to as “black-box” behavior since only the input and output variables are known to the developer. Therefore, the internal workings of the black box are still a mystery. Level 1–3 AVs, had recently caused several road accidents, that resulted in serious injuries or even fatalities. What caused the mishap? What problem in the driving system caused the accident? Some questions like these inevitably pose important ethical and security concerns and provide the motivation for explainable AI (XAI) systems. To improve road safety, the National Highway Traffic Safety Administration (NHTSA) of the US Department of Transportation has released a federal guideline on automated vehicle policy [103]. Therefore, both present-day and upcoming generations of autonomous vehicles must adhere to these new laws, and their intelligent driving systems must be explainable, understandable, transparent, and sufficiently secure.

The primary objective of the realization of XAI approaches in decision-making is to eliminate this opacity and make complex autonomous driving systems understandable and interpretable. Therefore, explainable planning can play an imperative role in helping users and enhancing their interactions with autonomous systems during complex decision-making processes. According to Sado et al. [104], the procedure may involve translating the agent’s plans into simple understandable formats and creating user interfaces that make this understanding easier, depending on the stakeholder. Furthermore, for a ready reference, the readers are highly recommended to look into the explainable planning relevant work which includes WHY-PLAN [105], XAI-PLAN [106], plan explicability and predictability, refinement-based planning [107], and plan explanation for model reconciliation.

### 4.2. Robust Decision-Making for Higher Level Autonomous Vehicles

Behavioral planning, local planning, and inter-vehicular communication for automation of lower levels are simpler when compared with the envisioned higher automation levels that involve: congested settings, many lanes populated with vehicles of varying speeds, roundabouts, intersections, crossings, etc. Conventional decision-making with a narrow environmental understanding and a short lifespan of decisions in terms of time and distance for critical maneuvers, compel the AVs to make myopic decisions, resulting in long-term erroneous decisions, injuries, fatalities, reduced traffic safety, efficiency, and comfort. When considering the planning horizon, there is a tradeoff between the accuracy of the perception and the time horizon. This means that the further the planning horizon is considered, the more imprecise the prediction of the environment. This trait is not a problem for a human driver. They are capable to integrate anticipatory behavior evaluating the current situation and evolving it with a more reactive behavior based on immediate maneuvers and perception. However, the current AVs are not capable of adapting the driving behavior to cope with uncertain dynamics due to limited perception and instant decision-making. Therefore, higher-level AVs require a self-driving system that incorporates a decision-making ability capable of dealing with unknown driving scenes and uncertainty.

The decision-making processes for current AVs are designed for relatively straightforward settings. It is not feasible to generalize the scenes, especially when making choices that affect the dynamics of several scenes within a scenario. Therefore, there is little interaction between the various decision-making instances. Accurate scenario-specific learning and decision-making present a significant challenge in the complex urban environment due to the rapid change in road dynamics. Furthermore, the restricted self-learning capabilities keep us from developing a fully developed and autonomous autopilot. Furthermore, it is still difficult to have the right computations at the right times and places. The decision-making instances of L5 AD need extensive and plentiful data to train the decision-making and learning models. The uncertainty and uninterpretability of the dynamic environment make it hard to achieve a full-fledged decision-making system for a higher level of autonomous vehicles.

### 4.3. Vehicle → Pedestrian Interaction

According to the World Health Organization (WHO), 1.35 million people died in traffic-related accidents in 2018, with pedestrians contributing to 21% of all fatalities [108]. The successful interaction between the AV and vulnerable road users (VRUs), particularly pedestrians, determines traffic safety in urban environments. Therefore, an understanding of these interactions is required to determine the behavioral requirements for L4 and L5 AVs.

The algorithms designed for decision-making must consider the interaction with other VRUs. However, the majority of the research works pay attention to decision-making under V2V interaction and rarely model vehicle-to-pedestrian (V2P) interaction. Since pedestrians are vulnerable traffic participants, making decisions with interaction with them is essential for safe driving. Future solution approaches for the decision-making system must integrate the contextual pedestrian perception, analysis and prediction of pedestrian actions, and modeling of multi-modal pedestrian behavior. As pedestrians are essentially multi-modal in nature and can feasibly travel various courses, AVs may have significant difficulties in accurately and reliably detecting and recognizing them. Besides, it is also challenging to detect, predict, and protect them. Pedestrians are challenging to perceive because of their changing physical attributes, appearance in a variety of situations, and various backgrounds, obstacles, and weather conditions. Therefore, the vehicle-based sensors may fail to recognize pedestrians even in ideal conditions, especially when the pedestrians are small, too far or too close to the AV, or partially obscured by other neighboring objects. To cope with these challenges the best suit of sensors, extended perception of the environment, Perception as a Service (PaaS), V2X, and intelligent sensor fusion solution approaches would also be required.

### 4.4. Collaborative Decision Making

As of now, AVs can drive more naturally due to the driving policies they have picked up from miles of actual driving environments. However, to further improving the autonomy of AVs; requires cooperation in every decision-making. AVs will be able to drive autonomously in various environments such as urban roads, highways, and freeways. An essential component of that is predicting the intentions of other AVs and coordinating maneuvers with them jointly. These kinds of cooperative maneuvers have the potential to significantly enhance traffic efficiency and safety. The ability to communicate and react to each other much faster and more precisely than humans ever could enable the possibility of collaborative, coordinated maneuvers among automated vehicles by leveraging the V2V and V2I communication technologies. Furthermore, collaborative maneuvering would guarantee safe and efficient navigation among multiple AVs, and intelligent strategies in situations like driving in an urban environment, multi-lane changing, overtaking, and entering/exiting highways would be taken to optimize traffic flow and minimize traffic congestion.

It is essential to note that the idea of collaborative decision-making may correspond to different decision-making situations instances and approaches in different AD use cases. This means that, in some circumstances, the decision to collaborate is simple because the vehicles just exchange information. However, in other circumstances such as; vehicle platooning—the decision to cooperate is followed by several other decisions. Now that the autonomous driving paradigm has quickly advanced to a more developed and mature stage, it is believed that it is important for the research community and industry to make a clear distinction between different types of collaborative decision-making mechanisms. Therefore, collaborative decision-making should be reflected in future decision-making frameworks which would resultantly improve maneuver planning. For a ready reference, the readers are highly recommended to look into detailed surveys on collaborative autonomous driving mechanisms [13,109].

### 4.5. Blended Approaches for Decision Making

Traditional approaches have clear levels, high scalability, and adjustability, with the advantage of breadth traversal, while learning-based approaches e.g., deep learning and reinforcement learning have a succinct structure and are ideal for processing particular scenarios with the advantage of deep traversal. Both methods have pros and cons, therefore, we believe that the fusion of different approaches at different stages of decision-making has the potential to achieve complementing benefits that should be considered in the future. For instance, to develop a highly intelligent decision-making system that integrates both breadth and depth, the top-level system may employ the FSM approach for quick decisions while the bottom level could train a distributed learning model based on particular scenarios for more complex decisions. However, among other challenges, one of the challenges would be ensuring the efficient and successful integration and fusion of different solution approaches. Along similar lines, Thurachen [32] proposed an algorithm to enable a vehicle to avoid a potential collision with a pedestrian crossing by integrating reinforcement learning and a well-defined rule-based technique. The proposed algorithm designed the reward function by considering safety, efficiency, and comfort. Experiments on the proposed algorithm were conducted in four different training scenarios in a simulated setting. The findings demonstrated that the algorithm has mastered the execution of longitudinal control when environmental uncertainty is introduced.

## 5. Conclusions

The autonomous vehicle is an all-encompassing intelligent system that integrates technology for environment understanding, path planning, decision-making, and motion control. The decision-making system in AVs plays an all-important role in executing safe and efficient maneuvers, therefore, how to build highly intelligent and trustworthy decision-making systems eventually becomes the primary research area. In this survey, we analyzed the solutions relevant to behavioral decision-making approaches in autonomous driving. Furthermore, also highlights the research gap, challenges, and potential directions for behavioral motion planning.

## Figures and Tables

**Table 1:** Space and Time Horizon for the Autonomous Driving Layers.

	Route Planning	Prediction	Decision Making	Generation	Deformation
**Space**	>100 m	>1 m <100 m	>10 m <100 m	>10 m <100 m	>0.5 m <10 ms
**Time**	>1 min <1 h	>1 s <1 min	>1 s <1 min	>1 s <1 min	>10 ms <1 s

**Table 2:** Comparison of the Classical Solution Approaches for Decision-Making.

Decision Making Approaches			Advantages	Disadvantages
**Classical** **Approaches**	**Rule** **Based**	Finite State Machine/ Hierarchical FSM	1. Good decision-making breadth. 2. Easy to understand and debug [49]. 3. Easy to implement and efficient in deterministic decision-making [16].	1. Results in poor explainability, maintainability, and scalability 2. Fully reliant on knowledge certainty and can not be generalized to unknown situations.
		Specific Rule Based	1. Simple, reliable, and easy to interpret [10]. 2. Applicability is superior in simple use cases such as lane change [10].	1. Cyclic reasoning and the exhaustive enumeration of rules leading to infinite loop and impact the computation time [3]. 2. Cannot maintain safe and efficient driving [10]. 3. Deemed applicable to the L-2 to L-4 and task-driven autonomous driving modes.
	**Planning** **Based**	Graph Based	1. Strong path searching capability in complex spaces [50]. 2. Implementation and formulation is usually simpler, more scalable, and modular [51].	Real-time performance is hard to achieve with graph-search algorithms [8].
		Optimization Based	1. Allows for a large action set to be used and optimized decisions can be generated [49,52]. 2. Interaction between different traffic participants can be modeled better [52].	1. Do not have the provision to consider uncertainty [49]. 2. Challenging to guarantee real-time performance and convergence [51]. 3. MPC requires a heavy computational load, due to its complex design and is unsuitable for high-speed autonomous driving and complex road trajectories [53].

**Table 3:** Comparison of coalitional game theory studies.

Coalitional Game Type	Ref.	Use Case/Application	Coalition of What?	Coalition Type	Cost Function Parameters/Metrics	Solution Concept	Simulator/Tool
Coalitional Formation	[65]	Multi-lane merging scenario	Connected AVs	Single Player, multi-player, grand & sub-coalition	Safety, Comfort, Efficiency	-	MATLAB/Simulink
Cooperative Coalitional	[70]	Cooperative lane change decision making.	Vehicles	-	Safety, Comfort, Efficiency	-	MATLAB/Simulink
Fuzzy Coalitional Game	[71]	Decision-making framework for CAVs at unsignalized intersection.	Connected AVs	Single Player & grand coalition	Driving safety, passing efficiency	Fuzzy Shapley value	MATLAB/Simulink
Coalitional Formation	[72]	Traffic optimization at multiple intersections.	Intersections	Dynamic	(1) Waiting time of vehicles; (2) number of vehicles passing in a certain time.	Nash equilibrium	NetLogo Simulator
Coalitional Graph	[73]	Platoon for intersection scenario.	Lanes	-	Throughput, the ratio of accidents	Nash equilibrium	-
Coalitional Formation	[74]	Platooning	Vehicles	Dynamic	Mean load per path, mean travel time	Shapley value	-
Coalitional Formation	[75]	Convoy driving on the highway.	Vehicles	Dynamic	-	-	Motes Devices, YAES simulator
Hedonic Coalition Formation	[77]	Platoon allocation and route planning for a shared transportation system in an urban environment.	Parked vehicles	-	Average number of the platoon, maximum tour duration, totally consumed energy	Nash stable	Java
Coalitional Formation	[76]	Spacing allocation method for platooning.	Vehicles	-	-	Shapley value, τ value & lexicographic value	-

## Data Availability

Not applicable.

## Abbreviations

The following abbreviations are used in this manuscript:

ADAS	Advanced Driver Assistance System
AIRL	Adversarial Inverse Reinforcement Learning
AD	Autonomous Driving
AI	Artificial Intelligence
AV	Autonomous Vehicle
CNN	Convolutional Neural Network
CAV	Connected and Autonomous Vehicle
CDD	Convoy Driving Device
CGT	Cooperative Game Theory
DARPA	Defense Advanced Research Projects Agency
FSM	Finite State Machine
GT	Game Theory
HMM	Hidden Markov Model
IL	Imitation Learning
LiDAR	Light Detection and Ranging
ML	Machine Learning
MPC	Model Predictive Control
MPD	Markov Decision Process
NCGT	Non-Cooperative Game Theory
NHTSA	National Highway Traffic Safety Administration
NTU	Non-Transferable Utility
POMDP	Partially Observable Markov Decision Process
RADAR	Radio Detection And Ranging
RAIL	Randomized Adversarial Imitation Learning
RRTs	Rapidly Explored Random Trees
SAE	Society of Automotive Engineers
TU	Transferable Utility
V2P	Vehicle-to-Pedestrian
V2V	Vehicle-to-Vehicle
V2VX	Vehicle-to-Everything
VRU	Vulnerable Road User
XAI	Explainable AI
